# Assessing the impact of seawater blockade on coastal lake degradation using Chironomidae larvae

**DOI:** 10.1038/s41598-025-93127-w

**Published:** 2025-03-24

**Authors:** Natalia Mrozińska, Katarzyna Glińska-Lewczuk, Sylwia Lew, Monika Szymańska-Walkiewicz, Krystian Obolewski

**Affiliations:** 1 Department of Hydrobiology, University of Kazimierz Wielki in Bydgoszcz, 85-090 Bydgoszcz, Poland; 2 Department of Water Resources and Climatology, University of Warmia and Mazury in Olsztyn, 10-719 Olsztyn, Poland; 3 Department of Microbiology and Mycology, University of Warmia and Mazury in Olsztyn, Oczapowskiego Str. 1a, 10-719 Olsztyn, Poland; 4 Department of Revitalization of Inland Waterways, University of Kazimierz Wielki in Bydgoszcz, 85-064 Bydgoszcz, Poland

**Keywords:** *Chironomus* sp., Anthropogenic lake degradation, Saline water intrusion, Hydrology regime, Flood gate, Baltic sea, Ecology, Ecology, Environmental sciences

## Abstract

Coastal ecosystems, such as lakes and lagoons, are unique and valuable water bodies whose proper functioning depends on hydrological connectivity with the sea or ocean. Human interventions, such as the construction of storm surge barriers, that block the periodic and free influx of seawater into lakes induce a permanent freshwater state. This study presents such disturbances, considered as environmental stressors, initiating changes in the assemblages of Chironomidae larvae inhabiting the bottom of Lake Jamno (southern coast of the Baltic Sea). Changes in the structure of this assemblages were assessed during a long-term study (2010–20), which considered two periods: a time of free seawater intrusion (FF) and seven years of blocked influx (BF). The findings indicate that, following the activation of storm surge barriers, the α-diversity of larvae consistently decreased throughout the lake. Concurrently, the density of Chironomidae larvae decreased by over 20%, although their biomass increased. In the last year of the study with functioning gates, the diversity of the studied insects was drastically reduced and was limited to only two genus: *Chironomus* sp. and *Procladius* sp., which serve as indicators of disturbances in aquatic ecosystems undergoing changes in line with deterministic chaos theory. The information provided indicates that periodic increases in salinity significantly affect the structure of Chironomidae larvae, though it should be considered as a component of several other parameters (EC, temperature, or nutrients).

## Introduction

Coastal zones occupy less than 15% of Earth’s land surface, yet coastal lakes and lagoons are among the most productive ecosystems globally^1^. The dynamics and duration of hydrological connectivity with the sea play a pivotal role in the functioning of these ecosystems. This connectivity simultaneously supports coastal ecosystems as nurseries, feeding grounds, and refuges for a diverse array of marine, freshwater, and terrestrial organisms^2,3^.

However, human activities are increasingly disrupting the hydrological connections of coastal ecosystems (such as bays, lagoons, and lakes) with the sea/ocean. The main motivation behind this interference is to provide flood protection through artificial modifications at the interface of these ecosystems, including the construction of storm surge barriers that hinder or completely block the influx of seawater. Often, an additional rationale for such interventions is expected increases in fishing efficiency and the desire to improve the abiotic conditions of sea-dependent ecosystems^4–8^. Unfortunately, the biological consequences of such changes are difficult to assess due to the overlapping influences of multiple local and global factors^9,10^.

As a result, artificial alterations in hydrological conditions can have significant consequences for ecosystems, especially in water bodies with ecological degradation features. This applies to most of the coastal lakes of the southern Baltic Sea, which struggle with increased nutrient concentrations^7,11,12^ and high primary production^13^ and that even, in the longer term, change due to intense secondary succession^14^. Therefore, prolonged isolation from seawater intrusion interferes with established ecological processes at various organizational levels in coastal ecosystems, forcing structural changes in biotic communities, including species extinction^14^. In view of this, benthic invertebrates serve as excellent indicators for tracking such changes, being highly sensitive to environmental alterations^15^. They form an integral part of the aquatic environment and play a crucial ecological role by maintaining high levels of interaction with the environment^16,17^. Hence, qualitative and quantitative changes in the structure of benthic fauna provide insights into the directions and pace of changes in water bodies^17,18^.

Among many representatives of benthic fauna, the Chironomidae family (Diptera) often dominates and is found in nearly all kinds of water bodies^19^. Members of this family play an important role in ecological studies^20,21^, primarily due to their resilience to poor habitat conditions and their ability to colonize new territories by occupying the ecological niches of other bottom-dwelling species^22^. Species within this family possess numerous features that make them 'ideal indicator organisms,' as they are easy to breed, are sensitive to many pollutants, have short life cycles, and are good indicators of the presence of toxic substances^23^. For these reasons, Chironomidae larvae are often treated as indicators of the health of aquatic ecosystems and as signals of the direction of changes these ecosystems are undergoing^21,22^. They can therefore be treated as bioindicators in understanding the mechanisms creating principles of deterministic chaos^24^.

Human-induced disturbances in coastal ecosystems imply a cascade of dynamic, unpredictable responses resulting from sensitivity to initial conditions (resilience to disturbances). The absence of periodic intrusion thus becomes a trigger to verify the assumptions of deterministic chaos theory^25,26^. This is particularly important in the context of developing rational programs for sustainable management of coastal areas to mitigate damages caused by catastrophic events^27,28^. Therefore, it is reasonable to assess the benefits and losses of planned or implemented actions in terms of ecosystem services in relation to provisioning, regulating, and supporting services^29^.

The aims of this study were to: (1) assess the long-term effects of isolating a coastal lake from seawater intrusion using the assemblages structure of Chironomidae larvae; (2) evaluate the impact of environmental predictors on individual Chironomidae larvae; (3) identify indicator type among the larvae associated with the transformation of an isolated lake ecosystem. The specific objectives aimed to verify the research hypothesis that prolonged isolation leads to a new ecological state in a coastal lake.

## Materials and methods

### Study area

Jamno Lake, situated in the central part of the southern Baltic coast in northern Poland, was the subject of this study. This shallow lagoon, covering 22.07 km^2^ with an average depth of 1.7 m, is segmented by the Podamirowski and Łabuski peninsulas into three distinct basins, each characterized by its riverine input. The western basin, Jamno Małe, receives water from the Strzeżnica River; the central basin, Jamno Centralne, from the Dzierżęcinka River; and the eastern basin, Jamno Osieckie, from the Unieście River. Historically, Jamno had periodic hydrological connections to the Baltic Sea through the Jamneński Channel, making it a transitional water body that showed the dynamic interface between freshwater and marine environments^8^. It has been observed that sudden but infrequent pulses characterized saline water inflows in autumn and winter. In contrast, in spring and summer, these inflows were milder but more frequent^30^. According to Majewski^31^, before the construction of sluice gates in the lake, its water level was about 0.1 m higher than that recorded in the Baltic Sea. Thus, it was estimated that the sea contributed approximately 16.1% to the lake’s total hydrological balance, amounting to nearly 30 million m^3^ of water. Schmidt^32^ noted that, in the years 1961–70, the number of days with inflows to the Jamneński Channel accounted for about a quarter of the year (~ 90 days).

**Fig. 1:**
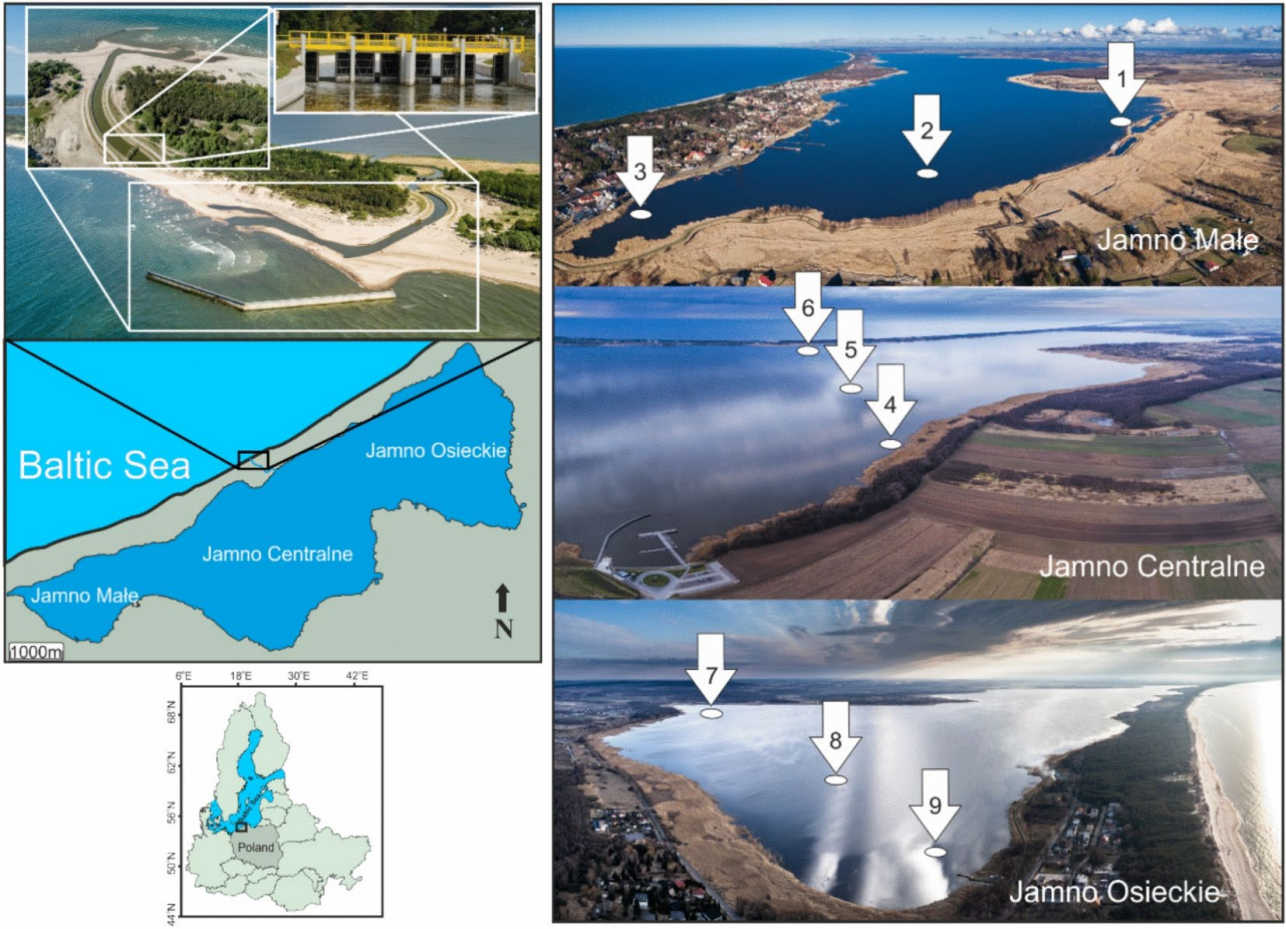
Location of storm gates on the Jamno Channel and the positioning of study sites across different basins of Lake Jamno (Photo created by Sky Drone).

Originally, the length of the Jamneński Channel was 500 m, but this was altered due to problems in maintaining its navigability, as well as the occurrence of backward-flooding caused by abundant freshwater flows from the land and seawater intrusion. Consequently, comprehensive engineering decisions were taken to modify the hydrological infrastructure. The channel was deepened by ~ 2 m to enhance water flow management and reinforced with concrete against dynamic hydrological forces. Additionally, technologically advanced floodgates were installed (Fig. 1), designed to provide precise control over the bidirectional water flow, thereby preventing uncontrolled marine inflow and safeguarding the inland hydrological regime. The structure has been operating since November 4, 2013 as a self-closing system, preventing the inflow of seawater into the lake.

### Sampling and sample processing

The research was conducted from 2016 to 2020 during the peak of the summer season (end of July to August), covering a period of complete blockade of Baltic Sea water intrusion into the lake. Macrozoobenthos samples were collected from nine sites located within the Małe Basin (sites 1–3), Centralne Basin (sites 4–6), and Osieckie Basin (sites 7–9), as shown in Fig. 1. All selected sampling sites were characterized by a muddy bottom and free from underwater vegetation. The depth was shallow and ranged from 1.0 to 1.2 m in the area of Małe Basin to 2.0 to 3.3 m in Centralne Basin.

Benthos samples were collected in triplicate at each site using an Ekman grab with a catching area of 225 cm^2^. On-site, samples were rinsed on a limnological sieve with a mesh size of 0.5 mm and preserved in 4% formaldehyde. In the laboratory, invertebrates were separated from sediments and identified to the lowest feasible taxonomic level, typically to the genus or species level, using morphological methods with a CX23 microscope (Olympus, Japan) and available keys for identifying Chironomidae larvae^33,34^. The biomass of identified organisms was measured as the mass of wet organisms preserved in formaldehyde, using an ALN 60G scale (AXIS, Poland) with a precision of 0.001 g. Results were expressed per 1 m^2^ of lake bottom area. Indicators describing the studied insect group included: species number (indiv.), total density (indiv. m^−2^), biomass (mg_ww_m^−2^), Shannon index (H’), and the density of the dominant Chironomidae family (indiv. m^−2^).

In parallel with the biological sampling, in-situ measurements of physicochemical water parameters were conducted at the same sites. A multiparameter probe AP-7000 (AquaRead, UK) was used to determine electrical conductivity (EC), pH, water oxygenation (DO%), salinity, and temperature. Water transparency was assessed using a Secchi disk (SD). Additionally, water samples were collected in 1L bottles for further laboratory analyses such as nutrient compounds (nitrate nitrogen and phosphate phosphorus) using a DR3900 spectrophotometer (HACH, USA) according to the methodology (APHA, 2017).

Simultaneously, data from 2010, before the construction of floodgates (free flow of seawater period [FF]), and from 2014 to 2015, shortly after their construction (blocked flow of seawater period [BF]), were used to trace long-term changes in the assemblages structure of Chironomidae larvae and habitat conditions. This information was supplemented by the database in Obolewski^8^.

### Data analysis

Based on density data for Chironomidae larvae collected during the study, a Bray–Curtis dissimilarity matrix was constructed. Variations in environmental predictors (physicochemical water parameters) and biological indicators (abundance, biomass, α-diversity) were analyzed between the period of free seawater intrusion (T + 0) and after its blockade (T + 1 to T + 7, excluding T + 5) using an ANOVA with Kruskal–Wallis (K–W) rank tests followed by Dunn’s *post-hoc* test at a significance level of p < 0.05. At this stage, the data were tested for normality using the Shapiro–Wilk test and for homoscedasticity using Levene’s test. To mitigate the impact of extreme values, variables (except pH) were log-transformed to log(x + 1)^33^. The dispersion of the Chironomidae larvael community structure was described using non-metric multidimensional scaling (nMDS) with CANOCO software version 5.10. The relationship between environmental parameters and Chironomidae larvae density was examined using redundancy analysis (RDA). Additionally, to illustrate statistical associations between identified Chironomidae larvae and environmental variables, a *t*-value biplot with Van Dobben circles was generated based on the RDA properties of physicochemical water parameters and larvae density. The *t*-value biplot is based on reduced-rank regression, linking multiple regressions between taxon abundances and specified descriptors within the model defined by RDA using Monte Carlo permutation tests (p < 0.05), (Manly, 2006). Van Dobben circles highlighted Chironomidae representatives as significantly responsive to the tested factor (*t*-value <|2|)^35^.

The QGIS software version 3^36^ was employed to visualize the spatial distribution of density, biomass, and α-diversity in the lake area over the years: before the intrusion blockade (2010) and after the construction of storm gates (2014–20).

## Results

### Environmental conditions

Parameters describing the physicochemical conditions of water showed significant differences during the study periods before (BF) and after (FF) floodgate construction as demonstrated by a one-way ANOVA (K–W = 28.93–54.55, p < 0.0001) (see Table 1).

**Table 1:** Water quality (mean ± SD) of Lake Jamno during periods of free seawater intrusion (FF) and blocking of marine inflows (BF), along with results from a one-way ANOVA test.

Parameters	Unit	FF	BF						*p*
		2010 T + 0	2014 T + 1	2015 T + 2	2016 T + 3	2018 T + 5	2019 T + 6	2020 T + 7	
Temperature	°C	24.2 ± 0.9	24.8 ± 0.4	15.7 ± 0.3	24.2 ± 0.7	23.9 ± 0.5	21.6 ± 0.6	19.9 ± 0.5	< 0.0001
Visibility	m	0.3 ± 0.2	0.2 ± 0.0	0.2 ± 0.0	0.2 ± 0.0	0.2 ± 0.0	0.3 ± 0.1	0.3 ± 0.1	< 0.0001
EC	µS cm^−1^	600 ± 149	224 ± 8	472 ± 9	410 ± 37	416 ± 14	645 ± 30	524 ± 21	< 0.0001
pH	–	8.24 ± 0.31	9.14 ± 0.20	8.76 ± 0.17	9.30 ± 0.09	9.92 ± 0.56	9.00 ± 0.23	8.57 ± 0.07	< 0.0001
DO	%	81 ± 10	124 ± 18	81 ± 9	139 ± 17	122 ± 13	131 ± 15	128 ± 13	< 0.0001
Salinity	PSU	0.31 ± 0.08	0.07 ± 0.00	0.26 ± 0.00	0.21 ± 0.02	0.20 ± 0.21	0.27 ± 0.02	0.23 ± 0.03	< 0.0001
PO₄^3−^-P	mg dm^−3^	0.34 ± 0.11	0.26 ± 0.04	0.12 ± 0.02	0.46 ± 0.37	0.18 ± 0.09	0.36 ± 0.68	0.15 ± 0.04	< 0.0001
NO_3_^−^- N	mg dm^−3^	1.03 ± 0.11	0.99 ± 0.37	0.92 ± 0.61	0.35 ± 0.05	0.27 ± 0.09	0.35 ± 0.08	0.27 ± 0.09	< 0.0001

Physical water conditions experienced notable fluctuations, particularly strong decreases in electrical conductivity (EC) from T + 0 to T + 1, and increases between T + 1 to T + 6 and T + 7 (Dunn’s *post-hoc* test, p < 0.0001). Water temperature significantly decreased in the initial years after the gate’s operation (from T + 1 to T + 2, p < 0.0001), and between T + 0 and T + 2, as well as T + 1 and T + 7 (both at p = 0.0001). Salinity, which demonstrated the greatest variability over the seven-year period, peaked before the gate construction (T + 0), then sharply decreased in the first year post-operation (T + 1) (p < 0.0001), later stabilizing at similar levels in subsequent years (Table 1). The dynamics of oxygen conditions also showed clear differences between T + 0 and T + 3, and T + 2 and T + 3 (both at p < 0.0001). Predictors related to trophic levels, such as phosphate phosphorus and nitrate nitrogen, significantly reduced their concentrations (K–W = 30.86 and 45.42, respectively). For NO_3_-N, substantial reductions were noted between T + 0 and T + 1 compared to T + 5, and from T + 1 to T + 7 (p < 0.0001), while the decline in PO_4_-P concentration was mainly between T + 0 and T + 2 (p = 0.0001).

### Structure of macrofauna

**Fig. 2:**
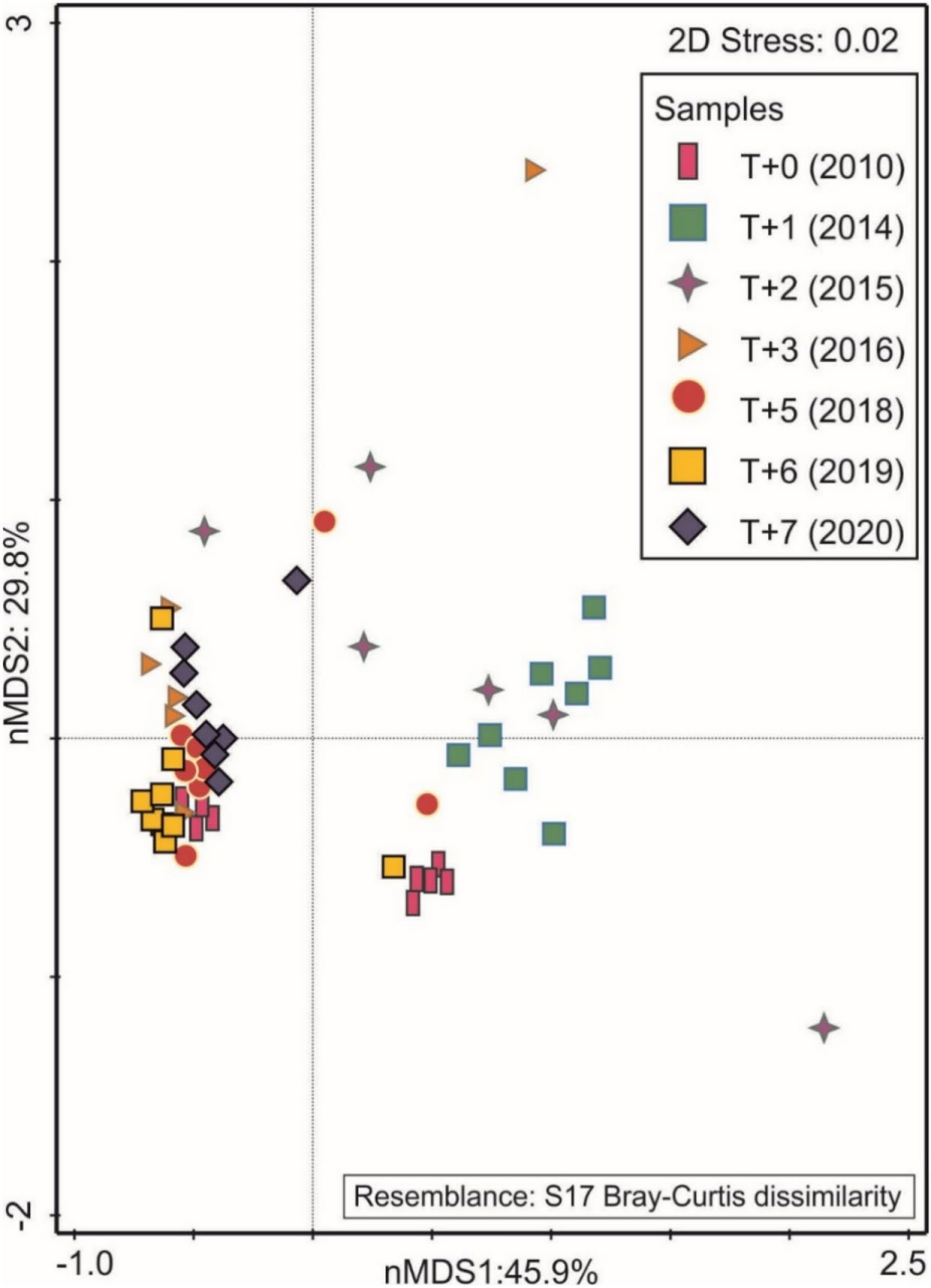
Multidimensional scaling (nMDS) ordination diagram showing the density structure of Chironomidae larvae in Lake Jamno during periods of free (FF) and blocked (BF) seawater inflow.

Multidimensional scaling (nMDS) based on qualitative and quantitative analysis of benthic fauna revealed differences in species density structure across various years. Together, both axes explained over 75% of the variance. The first axis primarily elucidated the abundance of larvae in the initial years analyzed (T + 0, T + 1, and T + 2), while the second axis did not exhibit significant associations (see Fig. 2). It is noteworthy that the results obtained have shown a marked convergence after three years of operating the storm gates. This trend is particularly pronounced in the most recent period studied (T + 6 and T + 7).

During the study in Lake Jamno, a total of six Chironomidae taxa were recorded. However, all taxa were observed only in the first year after the closure of the floodgates (T + 1), as shown in Table 2.

**Table 2:** Mean values (± SD) describing the Chironomidae larvae assemblages in Lake Jamno during periods of free intrusion (FF) and the blocking of seawater inflow (BF), along with results from one-way ANOVA analysis.

	FF	BF						*p*
	2010 T + 0	2014 T + 1	2015 T + 2	2016 T + 3	2018 T + 5	2019 T + 6	2020 T + 7	
Number of taxa	3	7	6	3	4	3	2	** < 0.003**
Total density (indiv. m^−2^)	503 ± 179	189 ± 112	86 ± 97	68 ± 113	165 ± 112	310 ± 208	110 ± 84	** < 0.0001**
Total biomass (g_ww_m^−2^)	0.35 ± 0.34	0.35 ± 0.25	0.64 ± 0.78	1.34 ± 2.11	2.37 ± 2.07	6.91 ± 5.53	2.57 ± 2.87	** < 0.002**
α-diversity (nat ind^−1^)	0.56	1.40	1.31	0.38	0.21	0.05	0.08	** < 0.001**
*Chironomus* sp. (indiv. m^-2^)	420 ± 196	866 ± 80	412 ± 57	61 ± 109	158 ± 116	286 ± 201	109 ± 84	< 0.0001
*Polypedilum nubeculosum* (indiv. m^−2^)	0.0	10 ± 17	2 ± 5	27 ± 5	0.0	0.0	2 ± 5	n.s
*Einfeldia carbonaria* (indiv. m^−2)^	0.0	13 ± 14	23 ± 14	0.0	2 ± 5	0.0	0.0	** < 0.001**
*Procladius* sp. (indiv. m^−2^)	49 ± 52	59 ± 45	13 ± 29	0.0	3 ± 10	2 ± 5	0.0	** < 0.0001**
*Cryptochironomus* sp. (indiv. m^−2^)	0.0	15 ± 21	2 ± 5	0.0	0.0	0.0	0.0	** < 0.002**
*Dicrotendipes* sp. (indiv. m^−2^)	0.0	2 ± 5	0.0	0.0	2 ± 5	0.0	0.0	n.s
Chironomidae n.det. (indiv. m^−2^)	35 ± 104	5 ± 11	5 ± 11	5 ± 7	0.0	21 ± 17	0.0	** < 0.002**

Significant values are in [bold].

The taxonomic diversity significantly varied over the course of the study (K–W = 20.20, p = 0.025), with marked differences particularly between T + 1 vs T + 3 and T + 1 vs T + 7 (Dunn’s *post-hoc* test, p < 0.006), as well as T + 1 vs T + 5 (p = 0.02). Significant disparities were observed in the density of invertebrate fauna (K–W = 30.87, p < 0.0001). The highest densities were recorded at T + 0, which significantly declined within the first year following the gate’s construction, achieving the lowest levels by T + 3 (p < 0.001). Variations were less pronounced between T + 0 and T + 2 (p = 0.0006), T + 0 and T + 7 (p = 0.005), and T + 3 and T + 6 (p < 0.05).

Total biomass of individuals fluctuated significantly (K–W = 20.97, p < 0.002) ranging between 0.35 and 6.91 g_ww_m^−2^, with the greatest differences noted between T + 0 vs T + 6 (p = 0.01), and T + 1 and T + 2 vs T + 6 (p < 0.03 in both cases). The diversity of Chironomidae larvae (Shannon index, H) showed significant oscillations (K–W = 23.46, p = 0.0007), with the highest values recorded at T + 1, while the lowest were at T + 7 (p = 0.004). Significant differences in α-diversity also involved the periods T + 1 vs T + 3 (p < 0.008) and T + 1 vs T + 6 (p = 0.01). *Chironomus* sp. was a representative of Chironomidae larvae, achieving the highest and most significant differences in density values across all periods (K–W = 31.09, p < 0.0001), particularly between T + 0 vs T + 2 and T + 3 (in both cases p < 0.001). Another significant taxon was *Procladius* sp. (K–W = 30.78, p < 0.0001), which experienced a sharp decline in abundance between T + 1 vs T + 3 and T + 7 (in both cases p = 0.0007). A slightly less significant drop in abundance was observed between T + 1 vs T + 5 and T + 6 (p = 0.005 and 0.004, respectively).

**Fig. 3:**
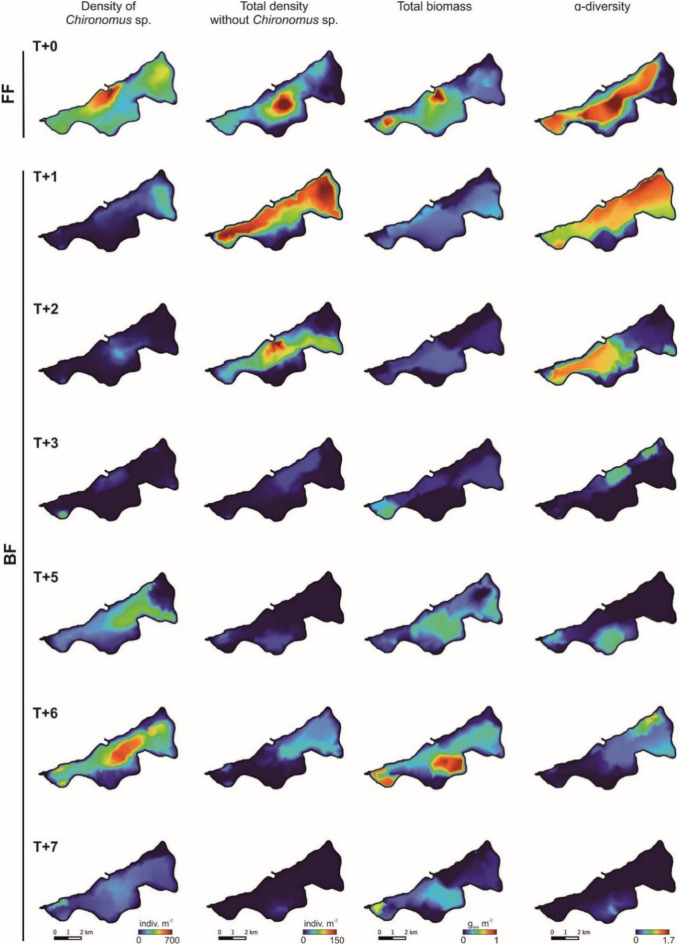
Spatial distribution of selected predictors (density of *Chironomus* sp., total density excluding *Chironomus* sp., total biomass, α-diversity) during periods of free seawater intrusion (FF) and blocked intrusion (BF) into Lake Jamno.

The spatial distribution of selected predictors indicated that, during periods with unrestricted seawater inflow into Lake Jamno, their values were higher than during the blockade period (BF) (Fig. 3). This was particularly evident in the basin of Jamno Centralne, treated as a hotspot for indicators describing benthic invertebrates. Following the activation of the storm gates, the density of the dominant *Chironomus* species decreased across all plots. After five to six years without intrusion, a slight increase in their abundance was noted in the central basin, followed by a sharp decline in the assemblages density of *Chironomus* sp. throughout the lake. In the case of the abundance of other identified Chironomidae larvae and α-diversity, a clear drop in values occurred three years after blocking the intrusion of Baltic waters across the entire Lake Jamno. This was a process that progressively intensified during the last period of the study in all basins (Fig. 3). The total biomass of the Chironomidae family, similar to other predictors, reached its highest values during the FF period in the central basin (the area of seawater inflow). Immediately after the gates were activated, the biomass significantly decreased throughout the lake; then, after five to six years, it slightly increased in the areas of Jamno Małe and Jamno Centralne. However, this increase was short-lived and not confirmed in T + 7, when a decrease in the biomass of the analyzed larvae occurred again in these basins (Fig. 3).

**Fig. 4:**
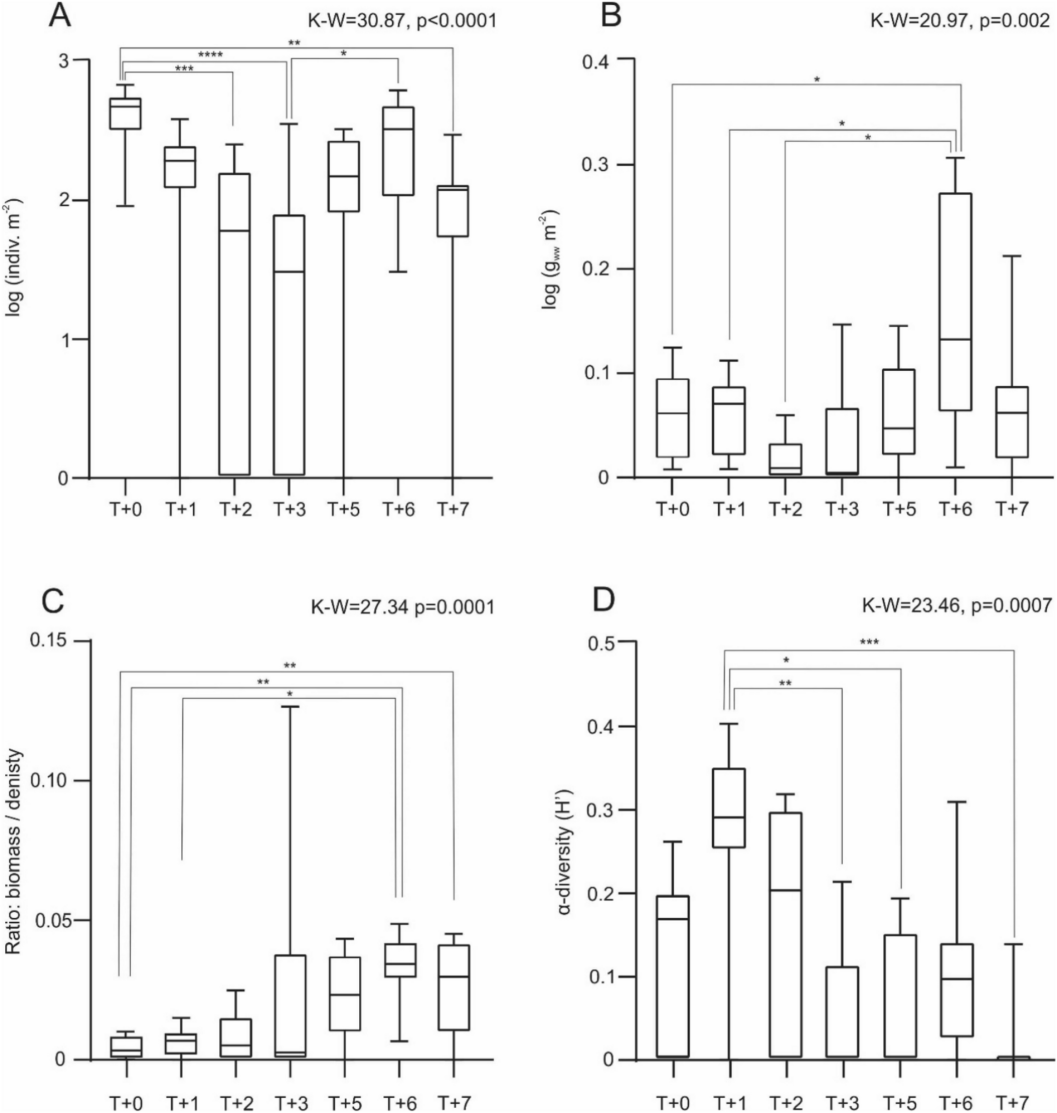
Average values (± SD of variables: density (**A**), biomass (**B**), biomass-to-density ratio (**C**), and α-diversity (**D**) during the study period were compared between conditions with free seawater intrusion (FF) and intrusion blocking (BF). Results included analysis of variance (Kruskal–Wallis test) and Dunn’s *post-hoc* tests. Significance levels were denoted as *p = 0.01; **p = 0.001; ***p = 0.0001; ****p < 0.0001.

A Kruskal–Wallis analysis extended with pairwise comparisons (Dunn’s *post-hoc* test) revealed statistically significant differences in density between the intervals T + 3 vs T + 5 (p = 0.01), T + 0 and T + 7 at p = 0.001, T + 0 and T + 2 (p = 0.0001), and between T + 0 and T + 3 (p < 0.0001) (Fig. 4A). For biomass, the results indicated statistically significant differences at p = 0.01 between the periods T + 0 vs T + 6, T + 1 vs T + 6, and T + 2 vs T + 6 (Fig. 4B). Biomass-to-density ratios showed significant statistical differences between T + 0 vs T + 7 and T + 0 vs T + 6, with p = 0.001, and between T + 1 vs T + 6 at p = 0.01 (Fig. 4C). Regarding α-diversity, significant statistical differences were found between T + 1 vs T + 5 (p = 0.01), T + 1 vs T + 3 (p = 0.001), and T + 1 vs T + 7 (p = 0.0001) (Fig. 4D).

**Fig. 5:**
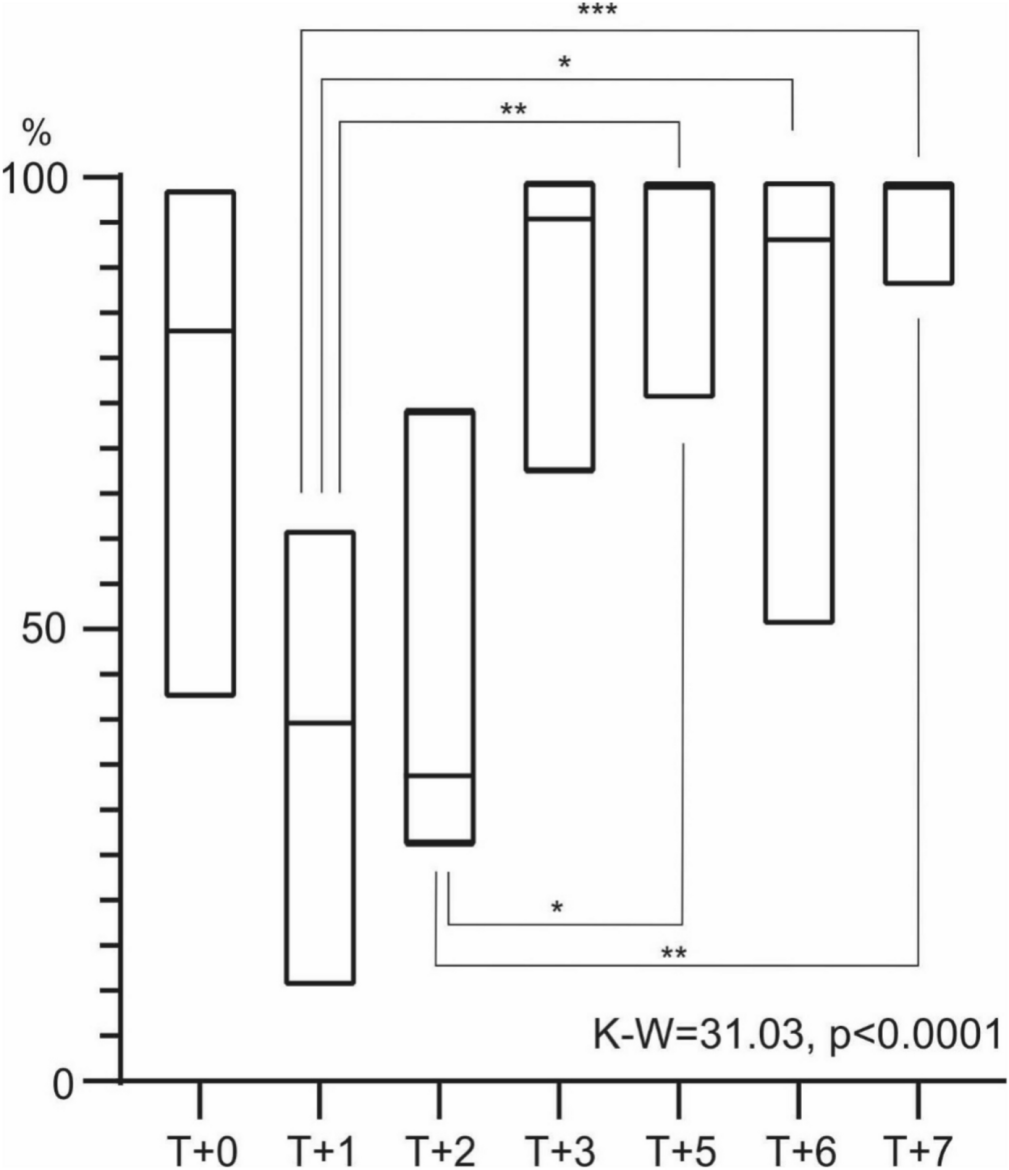
The share of *Chironomus* sp. among Chironomidae larvae (mean ± SD) during the FF and BF periods, along with results of the Kruskal–Wallis (K–W) and Dunn’s *post-hoc* tests (*p = 0.01; **p = 0.001; ***p = 0.0001).

Results of variance analysis for the dominance of the main component, *Chironomus* sp., in benthic fauna indicated statistically significant differences between the periods T + 1 and T + 6, as well as T + 2 and T + 5, at the p = 0.01 level. Significant differences were also observed between T + 1 and T + 5, and T + 2 and T + 7, at the p = 0.001 level. Furthermore, between the years T + 1 and T + 7, differences reached a significance level of p = 0.0001 (Fig. 5).

### Impact of environmental predictors on Chironomidae larvae assemblages

**Fig. 6:**
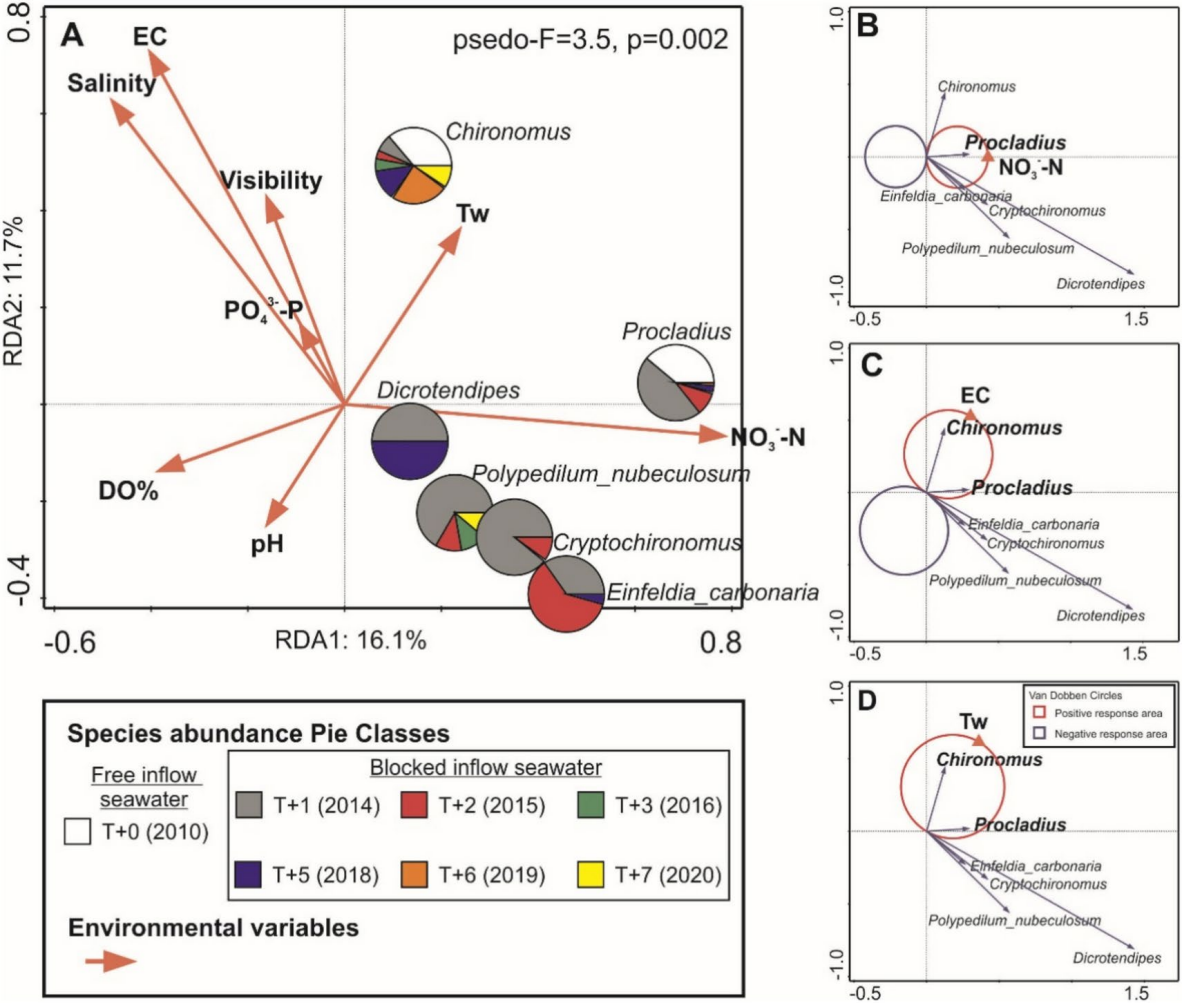
Results of redundancy analysis (RDA): (**A**) biplot of significant environmental variables and parameters of Chironomidae larvae (p < 0.05); and t-value biplots with Van Dobben circles based on the RDA of environmental variables and parameters of Chironomidae larvae: (**B**) Van Dobben circles for NO_3_-N; (**C**) Van Dobben circles for EC; and (**D**) Van Dobben circles for water temperature (Tw).

The relationships between environmental parameters and the structure of Chironomidae larvae were examined using Redundancy Analysis (RDA) and van Dobben circles. The final model accounted for 27.8% of the total variance in the assemblages structure of Chironomidae larvae. Among the key predictors of larvae structure were nitrate nitrogen concentration, electrical conductivity, and water temperature (Fig. 6A). The greatest impact on the first axis was observed for NO_3_-N and the density of *Procladius* sp., whereas for the second axis, salinity and conductivity, as well as the abundance of *Chironomus* sp., were influential. Additionally, this type of larvae achieved higher densities at lower values of DO concentration and water pH. At the same time, a negative response to high values of salinity and conductivity was noted among most Chironomidae larvae (excluding *Chironomu*s sp. and unidentified representatives of this family) (Fig. 6A).

Results from the *t-*value biplot (with van Dobben circles) indicated a positive response from *Procladius* sp. to increasing concentrations of NO_3_-N (Fig. 6B). As electrical conductivity and water temperature increased, both *Chironomus* sp. and *Procladius* sp. exhibited positive responses, as illustrated in Fig. 6C and D.

## Discussion

### Progressive ecological effects of isolating a coastal lake from seawater intrusions

Among the coastal ecosystems of the southern Baltic, Lake Jamno has long been considered one of the most vulnerable to degradation. The installation of storm gates in the lake, intended to safeguard local communities and infrastructure from flooding, shifted its brackish character to a freshwater state (Table 1). This artificial modification of Lake Jamno’s hydrological regime not only altered the structure of its biotic components but also affected the overall resilience of the ecosystem^37,38^.

The construction of hydraulic structures in coastal ecosystems exemplifies a dual-edged intervention: while such infrastructure can yield positive outcomes, including water level stabilization and flood protection for residential areas, it may simultaneously exert profound negative effects on aquatic ecosystem functioning and resident organisms. These impacts often arise from habitat alterations or the accumulation of pollutants^24^^–^^38^.

According to the literature, the ecological consequences of hydrotechnical interventions are dynamic, varying across temporal and spatial scales, and are influenced by climatic conditions and the trajectory of ecological succession^37,38,43,44^. We share the opinion of other authors, underscoring the necessity of assessing both the direction and magnitude of environmental change while balancing socio-economic benefits and losses.

Such an assessment requires the identification of reliable indicators capable of reflecting ecosystem health and responding to diverse stressors—physical, chemical, and biological^45^. Among the organisms particularly indicative of shifts in abiotic conditions and transformations within the benthic community are Chironomidae larvae. As a fundamental component of benthic fauna in coastal lagoons, their presence and composition serve as valuable bioindicators of environmental change^46,47^.

It has resulted not only in a transformation of abiotic conditions but also changes in the benthic biocenosis of such water bodies, predominantly represented by Chironomidae larvae^46,47^. Chironomidae larvae are a fundamental component of the benthic fauna found in coastal lagoons^46,48^. Early in the twentieth century, the potential of this family for water quality assessment was recognized^49,50^. Utilizing this observation, the results allowed for the verification of the formulated hypothesis and confirmed that isolating the coastal lake from pulsating marine intrusions significantly impacted the assemblage structure of Chironomidae larvae in the studied lake. The most tangible effect of blocking marine water intrusions was a decrease in the diversity of stress indicators used to assess the health of ecosystems (EcoHealth). Our study indicated that the absence of intrusions resulted in a drop in values for metrics such as density and α-diversity across the entire lake (Fig. 3). In terms of biomass, its values began to decrease in the first year after the gates’ construction (T + 1). At the same time, recovery occurred from T + 3, reaching peak values in T + 6.

This phenomenon can likely be attributed to the influx of nutrient-rich waters from river tributaries, namely the Dzierżęcinka and Strzeżenica, into the southern part of Lake Jamno, specifically affecting the Małe and Centralne lake basins (Fig. 3). However, this is not the sole factor contributing to the observed patterns. During the BF phase, an increase in the dominance of cyanobacteria was observed, which negatively impacted the functioning of the lake’s biocenosis. Notably, in 2019–2020, significant fish die-offs were reported^51^, primarily affecting benthivorous cyprinids^52^. This led to the elimination of predators that feed on Chironomidae larvae, further disrupting the lake’s ecosystem dynamics.

### Habitat quality change vs. assemblages condition of Chironomidae larvae

The absence of seawater intrusion resulted in changes across nearly all studied areas. This type of stressor appears to impact abiotic conditions first and foremost (Table 1). Subsequently, these changes triggered biological effects, representing indirect impacts that align with general theories on the importance of hydrological connectivity in dependent ecosystems^53^. The gate installation stops the effect of “pulsating intrusions” observed in transitional lakes such as Lake Jamno. In the case of this lake, the most significantly altered parameters were salinity and electrical conductivity (EC), which are directly linked to the intensity of marine water intrusions. It is well known that both predictors affect the composition and abundance of benthic organisms in lagoons^8,54^. For Lake Jamno, which was unique in its salinity and classified among brackish water lakes, even minor changes in salinity were identifiable stressors for Chironomidae larvae. The intensified lake degradation associated with the absence of intrusion created harsh conditions for aquatic organisms^8,55^. Significant changes may be led to increased mortality, inhibited activity, and restricted the reproductive ability of various assemblages. As a result of the blockage of hydrological connectivity in Lake Jamno, the density of Chironomidae larvae decreased by over 20%, while their biomass significantly increased. During the FF phase, the average mass of individual larvae was 7 × 10^4^ g_ww_ and consistently increased in the BF phase, reaching values from 7 × 10^3^ g_ww_ (T + 2) to 2 × 10^2^ g_ww_ (T + 7). This was due to the progressing simplification of the Chironomidae larvae assemblages in the BF phase, ultimately limited only to *Chironomus* sp. with decreasing significance of *Procladius* sp. Teske and Wooldridge^56^, in their studies, found that Chironomidae larvae are more abundant in ecosystems characterized by stable salinity levels. Thus, the forced, abrupt change in seawater volume in Lake Jamno was unequivocally a stress factor. However, it cannot be considered in isolation but rather as part of multiple interacting factors, such as temperature, oxygen concentration, and lake fertility^57^. The ordination analysis results (Fig. 6) indicated that, for *Chironomu*s sp., the influencing parameter was temperature, whereas for *Procladius* sp., it was the nitrate nitrogen concentration. Chironomidae’s distribution, abundance, and diversity are closely associated with temperature. The lowest water temperature values were recorded in 2015, which may be attributed to it being one of the cooler summer periods the studied years. As earlier results confirmed, *Chironomus* sp*.* is generally found in larger numbers in waters with medium to high temperatures^58,59^. In lakes, the distribution of Chironomidae is linked to thermal conditions. Temperature influences the physiology and distribution of this group of insects. It also modulates physiological processes and directly affects the modification of various limnological variables in lakes (e.g., nutrients and oxygen concentration)^59^. In the case of *Procladius* sp., it is a genus whose abundance may increase in the ongoing process of degradation. This genus is usually a potential biotic indicator of pollution in the lake, closely related to the increase in nutrient concentrations^60^. *Chironomus* and *Procladius* were the dominant genera in the studied water body. These taxa are most often found in the muddy sublittoral zone, especially in eutrophic lakes with unfavorable habitat conditions^59^.

### Which Chironomidae species optimally reflects the effects of enforced desalination in a coastal lake?

As pointed out by Obolewski^46^, the genus *Chironomus* was dominant in Lake Jamno prior to the construction of storm gates (pre-facility phase, FF), but after their operation (post-facility phase, BF), its density decreased nearly fourfold (Table 2). Given their ecological plasticity and adaptability to changes in environmental parameters and human interventions, which result in various alterations in aquatic ecosystems, *Chironomus* sp. can serve as an effective indicator of prevailing stressors^61^. Furthermore, representatives of this genus are among the largest Chironomidae larvae found in lowland lakes^50^, which may indicate a significant biomass increase in the last two years of the study, when the genus accounted for over 99% of the assemblages (Fig. 5). Classifications based on Chironomidae larvae have been developed as tools for assessing trophic status^62,63^ and lake acidification^64^. *Chironomus* sp. has also been used as an indicator of trophic conditions for decades^22,64,65^ due to its immense ecological tolerance to various stressors, whether caused by human activity or its absence^22^. For these reasons, *Chironomus* spp. may be an ideal indicator type for changes occurring in water bodies and serve as a useful tool in monitoring studies and planned conservation actions^61,62^. Research in Lake Jamno confirms the ubiquity of *Chironomus* spp. larvae and allows them to be treated as a universal indicator of salinity, oxygen and nutrient concentration changes in coastal ecosystem waters driven by human actions and global climatic processes. Given that the presented results should be considered a case study, it is essential to validate our observations through additional research that can confirm *Chironomus* sp. as an indicator of salinity changes in ecosystems.

## Conclusion

Coastal lakes are characterized by dynamic hydrological changes, heavily influenced by their connection to the sea. The construction of storm gates significantly affects these processes. Deterministic chaos theory is utilized to predict changes in dynamic systems. In cases of salinity changes in water bodies, this theory can be especially useful in estimating changes induced by enforced hydrological isolation from seawater inflow, altering parameters from initial conditions. These changes affect the structure of Chironomidae larvae, potentially leading to disruptions in lake succession or even to complete disappearance of Chironomidae larvae. The high ecological plasticity of Chironomidae larvae gives them a chance to adapt to the new conditions prevailing in the ecosystem. All these factors have complex ecological reactions and dependence on community and existing environmental conditions, suggesting that precise ecological assessments of the water body under study are necessary to make informed environmental management decisions. The taxon exhibiting the highest tolerance to severe environmental disturbances induced by the blockage of marine water intrusion was exclusively *Chironomus* spp. This genus was the sole representative of the benthic community capable of existing in the extreme aquatic conditions, particularly hypoxic periods and elevated water temperature. In the case of coastal lakes, the blockage of seawater inflows and the consequent changes in the ecosystem can be game-changing, providing a valuable reference point for understanding the direction and rate of change that an isolated coastal ecosystem may undergo.

## Data Availability

All biological data and physico-chemical parameters used in this article are available online on Figshare at the following link: https://figshare.com/articles/dataset/Biological_and_physicochemical_data_xlsx/27247518/1.

## References

[1] Obolewski, K. & Glińska-Lewczuk, K. Connectivity and complexity of coastal lakes as determinants for their restoration—a case study of the southern Baltic Sea. *Ecol. Eng.* **155**, 105948. 10.1016/j.ecoleng.2020.105948 (2020).

[2] Kennish, M. J. & Paerl, H. W. *Coastal Lagoons: Critical Habitats of Environmental Change* (CRC PRESS, 2010).

[3] Soares, N. et al. Urban effects in the sediment of an Intermittently Closed and Open Lagoon (ICOLL) in southeastern Brazil—a high-resolution study. *Environ. Monit. Assess.* **191**, 237. 10.1007/s10661-019-7358-7 (2019). 30903355 10.1007/s10661-019-7358-7

[4] Roy, K., Jablonski, D. & Valentine, J. W. Climate change, species range limits and body size in marine bivalves. *Ecol. Lett.* **4**, 366–370. 10.1046/j.1461-0248.2001.00236.x (2001).

[5] Dye, A. & Barros, F. Spatial patterns of macrofaunal assemblages in intermittently closed/open coastal lakes in New South Wales, Australia. *Estuar. Coast. Shelf Sci.* **64**, 357–371. 10.1016/j.ecss.2005.02.029 (2005).

[6] Gladstone, W., Hacking, N. & Owen, V. Effects of artificial openings of intermittently opening estuaries on macroinvertebrate assemblages of the entrance barrier. *Estuar. Coast. Shelf Sci.* **67**, 708–720. 10.1016/j.ecss.2006.01.008 (2006).

[7] Netto, S. A., Domingos, A. M. & Kurtz, M. Effects of artificial breaching of a temporarily open/closed estuary on benthic macroinvertebrates (Camacho lagoon, southern Brazil). *Estuar. Coast.* **35**, 1069–1081. 10.1007/s12237-012-9488-9 (2012).

[8] Obolewski, K., Glińska-Lewczuk, K. & Astel, A. Lost connectivity between a coastal lagoon and the sea—implication of floodgate closure for benthic macroinvertebrates. *Estuar. Coast Shelf Sci.* **211**(3), 1–13. 10.1016/j.ecss.2018.02.012 (2018).

[9] Bandyopadhyay, M. Effect of environmental fluctuation on a detritus based ecosystem. *J. Appl. Math. Comput.* **26**, 433–450. 10.1007/s12190-007-0029-9 (2008).

[10] Schallenberg, M., Larned, S. T., Hayward, S. & Arbuckle, C. Contrasting effects of managed opening regimes on water quality in two intermittently closed and open coastal lakes. *Estuar. Coast Shelf Sci.* **86**, 587–597. 10.1016/j.ecss.2009.11.001 (2010).

[11] Twomey, L. & Thompson, P. Nutrient limitation of phytoplankton in a seasonally open bar-built estuary: Wilson Inlet, Western Australia. *J. Phycol.* **37**(1), 16–29. 10.1046/j.1529-8817.1999.014012016.x (2001).

[12] Gobler, C. J., Cullins, L. A., Koch, F., Harder, T. M. & Krause, J. W. Influence of freshwater flow, ocean exchange, and seasonal cycles on phytoplankton—nutrient dynamics in a temporarily open estuary. *Estuar. Coast. Shelf Sci.* **65**(1–2), 275–288. 10.1016/j.ecss.2005.05.016 (2005).

[13] Szymańska-Walkiewicz, M., Matela, M. & Obolewski, K. Patterns of effects of land-use structure on lake water quality in coastal lake catchments of the southern Baltic Sea. *Ecohydrol. Hydrobiol.* **24**, 447–458. 10.1016/j.ecohyd.2023.07.004 (2024).

[14] Bate, G. A review of information on temporarily open/closed estuaries in the warm and cool temperate biogeographic regions of South Africa, with particular emphasis on the influence of river flow on these systems, Pretoria 192–214 (2007).

[15] Dufrêne, M. & Legendre, P. Species assemblages and indicator species: the need for a flexible asymmetrical approach. *Ecol. Monogr.* **67**, 345–366. 10.1890/0012-9615(1997)067[0345:SAAIST]2.0.CO (1997).

[16] Anderson, N. H. & Sedell, J. R. Detritus processing by macroinvertebrates in some ecosystems. *Annu. Rev. Entomol.* **24**, 357–377. 10.1146/annurev.en.24.010179.002031 (1979).

[17] Emere, M. C. & Nasiru, C. E. Macroinvertebrates as Indicators of the Water Quality of an Urbanized Stream, Kaduna Nigeria. *Nat. Sci.* **6**, 1–7 (2008).

[18] Marques, M. J., Martinez-Conde, E. & Rovira, J. V. Effects of zinc and lead mining on the benthic macroinvertebrate fauna of a fluvial ecosystem. *Water Air Soil Poll.* **148**, 363–388. 10.1023/A:1025411932330 (2003).

[19] Cranston, P. S. & Hare, L. Conochironomus Freeman: an Afro-Australian *Chironomini* genus revised (Diptera: Chironomidae). *Syst. Entomol.* **20**, 247–264. 10.1111/j.1365-3113.1995.tb00096.x (1995).

[20] Sharley, D. J., Pettigrove, V. & Parsons, Y. M. Molecular identification of *Chironomus* spp. (Diptera) for biomonitoring of aquatic ecosystems. *Aust. J. Entomol.* **43**(4), 359–365. 10.1111/j.1440-6055.2004.00417.x (2004).

[21] Grebenjuk, L. P. & Tomilina, I. I. Morphological deformations of hard-chitinized mouthpart structures in larvae of the genus *Chironomus* (Diptera, Chironomidae) as the index of organic pollution in freshwater ecosystems. *Inland Water Biol.* **7**, 273–285. 10.1134/S1995082914030092 (2014).

[22] Lindegaard C. Classification of water-bodies and pollution. In *The Chironomidae. The Biology and Ecology of Non-Biting Midges* (eds. Armitage, P. et al.) 385–404 (Chapman & Hall, 1995).

[23] Callaghan, A., Fisher, T. C., Grosso, A., Holloway, G. J. & Crane, M. Effect of temperature and pirimiphos methyl on biochemical biomarkers in *Chironomus riparius* Meigen. *Ecotoxicol. Environ. Saf.* **52**, 128–133. 10.1006/eesa.2002.2160 (2002). 12061829 10.1006/eesa.2002.2160

[24] Williams, N. et al. Response of Chironomidae to environmental disturbances in a high mountain. *Quat. Res.* **92**(2), 273–287. 10.1017/qua.2019.5 (2019).

[25] Toker, D., Sommer, F. T. & D’Esposito, M. A simple method for detecting chaos in nature. *Commun. Biol.* **3**, 11. 10.1038/s42003-019-0715-9 (2020). 31909203 10.1038/s42003-019-0715-9 PMC6941982

[26] Moiseenko, T. I. Evolution of ecosystems under an anthropogenic load: from disorganization to self-organization. *Geochem. Int.* **58**, 1083–1091. 10.1134/S0016702920100110 (2020).

[27] Ombadi, M., Nguyen, P., Sorooshian, S. & Hsu, K.-L. Complexity of hydrologic basins: a chaotic dynamics perspective. *J. Hydrol.* **597**, 126222. 10.1016/j.jhydrol.2021.126222 (2021).

[28] Jayawardena, A. W. *Environmental and Hydrological Systems Modelling* (CRC Press, 2013).

[29] Heino, J. et al. Lakes in the era of global change: moving beyond single-lake thinking in maintaining biodiversity and ecosystem services. *Biol. Rev.* **96**, 89–106. 10.1111/brv.12647 (2021). 32869448 10.1111/brv.12647

[30] Heese, T. (2012). Ekspertyza w zakresie oceny wpływu przedsięwzięcia na cele ochrony wód w rozumieniu art. 4.1.w związku z artykułem 4.7. Ramowej Dyrektywy Wodnej dla przedsięwzięcia: Etap I – Modernizacja i odbudowa brzegów morskich, ochrona Mierzei Jamneńskiej, Koszalin (in Polish)

[31] Majewski, A. Charakterystyka hydrologiczna estuariowych wód u polskiego wybrzeża. *Prace PIHM* **105**, 3–37 (1972).

[32] Szmidt, K. Hydrology of the Baltic lakes, with particular emphasis on the lake Jamno. *Stud. Oceanol. Mater.* **3**, 1–25 (1973).

[33] Andersen, T., Cranston, P. S. & Epler, J. H. Chironomidae of the holoarctic region: keys and diagnoses. Part 1—larvae. *Syst. Entomol.* **2013**, 89 (2013).

[34] Romaniszyn, W. Klucze do oznaczania owadów Polski. In *XXVIII Muchówki – Diptera, larwy. Zeszyt 14a. Ochotkowate- Tendipedidae* (PWN Waraw, 1958).

[35] Ter Braak C. J. F. & Smilauer P. CANOCO reference manual and Canodraw for windows user’s guide: software for canonical community ordination (Version 4.5) Biometris; Wageningen, The Netherlands: CANOCO software version 5.10 (2002).

[36] Ter Braak, C. J. E. & Looman, C. W. N. Biplots in reduced-rank regression. *Biota. J.* **36**, 983–1003. 10.1002/bimj.4710360812 (1994).

[37] QGIS Development Team, QGIS Geographic Information System. Open Source Geospatial Foundation Project. http://qgis.osgeo.org (2023).

[38] Gates, A. R. et al. Ecological role of an offshore industry artificial structure. *Front. Mar. Sci.* **6**, 675. 10.3389/fmars.2019.00675 (2019).

[39] Biede, V. et al. Short-term response of deep-water benthic megafauna to installation of a pipeline over a depth gradient on the Angolan Slope. *Front. Mar. Sci.* **2022**, 14. 10.3389/fmars.2022.880453 (2022).

[40] Bell, N. & Smith, J. Coral growing on North Sea oil rigs. *Nature* **402**(6762), 601–601. 10.1038/45127 (1999).10604464

[41] Todd, V. L. G., Warley, J. C. & Todd, I. B. Meals on wheels? A decade of megafaunal visual and acoustic observations from offshore oil & gas rigs and platforms in the North and Irish Seas. *PLos One* **11**(4), e0153320. 10.1371/journal.pone.0153320 (2016). 27078153 10.1371/journal.pone.0153320 PMC4831756

[42] Claisse, J. T. et al. Oil platforms off California are among the most productive marine fish habitats globally. *Proc. Natl. Acad. Sci.* **111**(43), 15462–15467. 10.1073/pnas.1411477111 (2014). 25313050 10.1073/pnas.1411477111 PMC4217422

[43] Cordes, E. et al. Environmental impacts of the deep-water oil and gas industry: a review to guide management strategies. *Front. Environ. Sci.* **4**, 58. 10.3389/fenvs.2016.00058 (2016).

[44] Fujii, T. Temporal variation in environmental conditions and the structure of fish assemblages around an offshore oil platform in the North Sea. *Mar. Environ. Res.* **108**, 69–82. 10.1016/j.marenvres.2015.03.013 (2015). 25965149 10.1016/j.marenvres.2015.03.013

[45] Choiński, A., Gogołek, A. & Mrugalski, T. Perennial changes of water chemistry in the Jamno lake. *Limnol. Rev.* **1**, 107–115 (1998).

[46] Obolewski, K. Using macrozoobenthos to assess the ecological condition of the estuary Jamno Lake. *Ochrona Środowiska* **31**(2), 17–24 (2009).

[47] Cieśliński, R. Changes in salinity and water level of the Lake Jamno resulting from the construction of storm gates. *Environ. Prot. Eng.* **16**(4), 517–539. 10.17512/ios.2016.4.7 (2017).

[48] Malej, J. Fauna denna w zanieczyszczonym estuarium. *Stud. i Mat. MIR* **13**, 7–83 (1974).

[49] Armitage, P. D. Chironomidae as food. In *The Chironomidae: The Biology and Ecology of Non-Biting Midges* (eds. Armitage, P. et al.) 425–428 (Chapman & Hall, 1995).

[50] Shadrin, N. V., Anufriieva, E. V., Belyakov, V. P. & Bazhora, A. I. Chironomidae larvae in hypersaline waters of the Crimea: diversity, distribution, abundance and production. *Eur. Zool. J.* **84**(1), 61–72. 10.1080/11250003.2016.1273974 (2017).

[51] Thienemann, A. D. Zusammenhang zwischen dem Sauerstoffgehalt des Tiefenwassers und der Zusammensetzung der Tiefenfauna unserer Seen. *Int. Rev. ges. Hydrobiol. Hydrogr.* **6**, 243–249 (1913).

[52] https://gk24.pl/badania-jeziora-jamno-dlaczego-tysiace-ryb-wymarlo/ar/c1-14290421 (2024).

[53] Kujawa, R. Ichthyofauna. In *The Ecological Status of the Southern Baltic Coastal Lakes* (ed. Obolewski, K. ) 202–203 (PWN Warsaw, 2017).

[54] Ward, J. V., Tockner, K., Arscott, D. B. & Claret, C. Riverine landscape diversity. *Freshw. Biol.* **47**, 517–539. 10.1046/j.1365-2427.2002.00893.x (2002).

[55] Kjerfve, B. Coastal lagoons. In *Coastal Lagoon Processes, Elsevier Oceanographic Series* (ed. Kjerfve, B.) 1–8 (Elsevier, 1994).

[56] Teske, P. R. & Wooldridge, T. H. What limits the distribution of subtidal macrobenthos in permanently open and temporarily open/closed South African estuaries? Salinity vs sediment particle size. *Estuar. Coast. Shelf Sci.* **57**(1–2), 225–238. 10.1016/S0272-7714(02)00347-5 (2003).

[57] Shadrin, N. V., Belyakov, V. P., Bazhora, A. I. & Anufriieva, E. V. Does salinity affect body proportions and “size/mass” ratios of highly halotolerant *Baeotendipes noctivagus* larvae (Diptera, Chironomidae)?. *Oceanol. Hydrobiol. St.* **48**(4), 305–315. 10.2478/ohs-2019-0028 (2019).

[58] Larocque, I., Pienitz, R. & Rolland, N. Factors indicating the distribution of chironomids in lakes distributed along a latitudinal gradient in northwestern Quebec, Canada. *Can. J. Fish. Aquat Sci.* **63**, 1286–1297 (2006).

[59] Eggermont, H. & Heiri, O. The chironomid-temperature relationship: expression in nature and palaeo environmental implications. *Biol. Rev.* **87**(2), 430–456. 10.1111/j.1469-185X.2011.00206.x (2011). 22032243 10.1111/j.1469-185X.2011.00206.x

[60] Coa, Y. et al. Combined effects of nutrients and trace metals on chironomid composition and morphology in a heavily polluted lake in central China since the early 20th century. *Hydrobiologia* **779**, 147–159. 10.1007/s10750-016-2810-y (2016).

[61] Péry, A. R. R., Mons, R. & Garric, J. Modelling of the life cycle of *Chironomus* species using an energy-based model. *Chemosphere* **59**(2), 247–253. 10.1016/j.chemosphere.2004.11.083 (2005). 15722096 10.1016/j.chemosphere.2004.11.083

[62] Saether, O. A. Chironomid communities as water quality indicators. *Ecography* **2**, 65–74. 10.1111/j.1600-0587.1979.tb00683.x (1979).

[63] Wiederholm, T. The ecology and aquatic insects. In *Response of Aquatic Insects to Environmental Pollution* (eds. Res, V. H. & Rosenberg, D. M.) 508–557 (Praeger Publ. New York, 1984).

[64] Raddum, G. R. & Sæther, O. A. Chironomid communities in Norwegian lakes with different degrees of acidification. *Verh. Int. Ver. Theor. Angew. Limnol.* **21**(1), 399–405. 10.1080/03680770.1980.11897014 (1981).

[65] Thienemann, A. Seetypen. *Naturwissenschaften* **9**, 343–346. 10.1007/BF01487893 (1921).

